# Massage

**Published:** 1920-02-28

**Authors:** 


### THE LIFE OF A NURSE: ITS MORAL AND MATERIAL ADVANTAGES.

### Massage.

Massage is a branch of the nursing profession which is becoming more prominent and more vital every day. A well-trained masseuse, especially if she has a general training behind her, can make a more comfortable income without undue exertion than is possible in any other branch of the exacting but badly-paid nursing profession.

Massage has only recently been fully appreciated by the medical profession in this country, and at present is "booming"—to use, a very unprofessional term. It is the long, tedious cases which form the regular income of the private practice, and take up a good deal of the time of all masseuses. But the monotony and hopelessness of these are more than compensated by the few acute cases, where one has the joy of seeing the useless limbs gradually recover bone and strength, the stiff fingers relax and grasp better every day, or the one-sided smile slowly but surely spread all over the face. Sometimes there comes a moment of wild excitement, after weeks, even months, of apparently hopeless work on a paralysed leg—at the discovery of a slight, almost imperceptible movement of the big toe!—the beginning of a recovery which will go on, fast or slowly, from that moment.

Massage has been a tremendous factor in the nursing of our brave soldiers in this war. Hundreds of men can testify to its benefits. It has made all the difference between being a helpless burden and earning his own living to many a discharged hero. The miracle of the healing of the withered arm is being repeated here in our hospitals every day—and it is no less a miracle that it is wrought by means which we are beginning to understand.

### Cost and Conditions of Training.

The massage course of instruction takes six months, and most training-schools will train certificated nurses free of charge in exchange for a year or so of work in the hospitals. Probationers are also trained at the end of two or three years' service. If one has a little capital it is more satisfactory to take the course as an external student and give one's whole time to it. The cost is from £50 to £80, and there is usually some arrangement for boarding the students who live far away. This is much less strain on the health than working in hospital and studying at the same time, and one is ready to start practice as soon as the examination is passed. Health is very important in a masseuse: the actual work requires a good deal of physical strength and stamina. A nervous, highly-strung girl may do good massage, but she is apt to give too much of her own vitality to the work; and sooner or later nature presents the bill, and she has to give up.

### Qualities and Qualifications.

The perfect masseuse has perfect health, although, like all nurses, she is none the worse for knowing a little what it feels like to be ill. She should be fairly tall and broad, so as to have complete physical control over all the patients without effort. She needs plenty of tact and not too ready a tongue. When it is remembered that she is screened off or shut in with the patient for half to one hour at a time it is easily seen how much harm may be done by indiscreet conversation. The mental effect of a woman thoroughly healthy in mind and body is often as beneficial to the patient as the manual treatment. The massage student must also have a fairly retentive memory. One cannot hope to restore muscles and nerves to their proper function unless one knows very minutely what and where they are. In addition to this, one needs a very exact knowledge of all the principal diseases usually treated by massage—their causes, symptoms and the treatment indicated by these symptoms. The various movements and manipulations must be acquired by hours of patient practice—and, still worse, must be clearly set forth on paper when required.

### The Examinations.

The first feeling on being confronted with the amount of knowledge one is expected to absorb in six months is that it is simply impossible and absolutely hopeless to try. Then comes the remembrance that scores of others, not noticeably more brilliant than we feel ourselves, have done it, and soon the fascination of study has seized all one's thoughts, and the days fly by and the examination is here before one can believe it possible. Most training-schools have their own examination, for which certificates are given, but the standard is the examination of the Incorporated Society of Trained Masseuses. For this there are two papers—one anatomy, three hours; and one massage, in theory, two and a half hours; and a month later an anatomy *viva voce* and an hour's practical massage on a live model—twenty minutes each for three different examinations. Or the subjects may be taken separately—anatomy on one occasion and massage on another. Every student after the examination is quite sure she has failed in one or all the tests, until the anxiously-awaited result tells her that her fears were groundless and she is now a qualified masseuse. And then how soon she begins to inquire what she can take next!

### Prospects after Certification.

Now that the need for masseuses is so urgent most girls take good posts at once; the more ambitious ones return to their books once more and work for the Swedish Remedial Exercise examinations and the Teachers' Certificate. It is wise to take a three-months' course in medical electricity, as that is often required in conjunction with massage, and is comparatively easy to master. Hospitals usually include it in the period of service. A private masseuse depends entirely on the connection she has, but it is usually quite easy to get enough work and sufficiently good fees to live quite comfortably without overwork. In hospital the usual rate of remuneration is from 2½ to 3 guineas, with or without board, as arranged. Any one holding the I.S.T.M. certificate can join the Almeric Paget Corps—both being careful to maintain a high standard and taking all the best posts, military and other.

In civil hospitals the massage sister gets from £80 to £100, with board and uniform. The hours are not long as the work is strenuous, six to eight hours' massage is usually considered a day's work, and every week-end is free, so that the masseuse can keep in touch with the world in a manner which is quite impossible to an ordinary hospital nurse.

